# Obstacles along the path of women enterprises in Africa: A case study of Ogotun women in Ekiti state, Nigeria

**DOI:** 10.1016/j.heliyon.2021.e07593

**Published:** 2021-07-16

**Authors:** Emmanuel F. Jaiyeola, Mercy Modupe Adeyeye

**Affiliations:** a College of Education, Washington State University, Pullman, WA, USA; b Department of Entrepreneurship and Business Studies, and SDG 5, Federal University of Technology, Minna, Nigeria

**Keywords:** Gender entrepreneurs, Women, Colonialism, Patriarchy, Nigeria, Africa

## Abstract

African women have a history of entrepreneurship dating back to the pre-colonial era, but in this 21st century, women in Africa are assessed by the World Bank to be at the lower deck of economic breakthrough in comparison to men. Although both men and women operate in the same business climate, women are usually poorer and are harder hit by adverse economic situations. Oftentimes, they self-develop their businesses and skills; they also seek, create and self-fund opportunities, but these efforts are usually met with huge obstacles and barriers. Therefore, this study aims to identify some of the obstacles and barriers plaguing the success and growth of women entrepreneurial activities and proffer solutions that could ameliorate the problems. The study uses a qualitative case study research design to uncover the experiences of women in a rural community of southwestern Nigeria. The data for analysis were collected through twenty in-depth interviews, two participants observations, and four focus group discussions. Some documents were analyzed to triangulate the data sources to ensure credibility. Findings include obstacles such as a traditional patriarchal culture that inhibits women from achieving personal development, complete subordination to male domination, colonial vestiges that gendered entrepreneurship in Africa, and lack of support by male-dominated government. The implication of these is that women continue to struggle to develop their entrepreneurial activities without much breakthrough. They remain economically impoverished and suffer greatly to care for themselves and their families, and to be part of social development. The study recommends that relevant organizations and government continue to work to develop strategies to remove these barriers.

## 1 Introduction

### 1.1 Precolonial history of women in entrepreneurship

The pre-colonial times in Nigerians, just like in many other Africans countries, people enjoyed communal living. The men and women in the communities, in their traditional settings, worked together in different roles which were not necessarily gendered but classified according to age hierarchy. They had the cultural understanding that their roles were complementary and with one desire, the benefit of the community (Ako-Nai, 2013). There was no segregation because whatever task (economic activity, domestic work, or community service) accomplished was for communal benefits (Jaiyeola and Aladegbola, 2020).

However, colonization redesigned the socio-cultural and economic structures that predated the colonial era in Nigeria. It also left a legacy of different social classes rooted in masculinity, and this bred social and economic inequalities in the post-colonial era. Additionally, patriarchy was manifested in every area (in employment, access to resources, governance, domestic and corporate roles), constraining both women and girls to a marginalized lifestyle. Women and girls were ‘subjects’ of the social construction of inferiority to boys and men (Nealon and Giroux, 2011; Leon-Guerrero, 2009) that placed them in an unequal status with men and boys. Presently, women face challenges of glass ceilings at workplaces, inadequate support in entrepreneurial activities; poor political representation; deprivation of reproductive, legal and inheritance rights; and limited access to education (Olawoye et al., 2004). However, recent studies and reports show that entrepreneurial activities are increasing, and more women are participating than before (Tende, 2016), for example, there are more women in the food production business in sub-Saharan Africa producing about 80 percent of the food in the region (Ali and Ali, 2013). Despite this, women-owned businesses are not financially viable; they record low on profit and are more likely to fail eventually (Tende, 2016; World Bank, 2019). This is another dimension to the disparity existing between men and women. Although this problem appears global, the intensity is felt more in regions of the world with severe economic hardship like the African continent. It is known that Africa's population is at parity with women and men, but women are less productive in the economy due to socio-cultural barriers (Kitching and Woldie, 2004). This is likely to continue to inhibit the overall living standards of women, especially, and the overall economic development of the nation. Therefore, the objective of this paper is to uncover and explain some of the obstacles that inhibit women entrepreneurship in Africa and propose solutions that could ameliorate the problems. In answering to the overarching research question: What are the experiences of rural women in fostering their business activities under African cultural and traditional practices? the study uses the case study of some women in Ogotun, a rural town in southwestern Nigeria, as the unit of analysis. While the result is not generalizable, the process is transferable to other parts of Africa because of common features like history, culture, and practice. This may produce same results or come up with more findings.

## 2 Literature review

### 2.1 Women in Africa, entrepreneurship in Africa, genderized entrepreneurship in Africa

The British colonizers took over the rulership in Nigeria in 1884 and had a significant effect on the pre-colonial socio-economic relationships (Okoronkwo-Chukwu, 2013). Women's roles and power were withdrawn or reduced, especially from economic participation, and were confined solely to their homes; this was the situation in all the colonies of the British and French in Africa (Okoronkwo-Chukwu, 2013; Jaiyeola and Aladegbola, 2020). Men were given the foremost privilege to western education thus creating strongly established class structures as men became the new working class while women, who could not work, had to depend on men for money (Adu, 2013). “Women thus began to carry the extra burden of colonization and inequality due to such marginalization and segregation” (Jaiyeola and Aladegbola, 2020).

Furthermore, the colonizers divided the Nigerian society into two categories along the lines of the businesses people engaged in. Since women were withdrawn from commercial activities, they were limited to cultivating food crops for family consumption. This brought in very low income because almost no one bought food then (Ako-Nai, 2013). The British colonizers made men to farm cash crops, such as cocoa, rubber, cotton, and coffee, and these were exported to England for industrialization. They also introduced trades that were solely for their benefit into Nigeria and further marginalized women. With this disruption of the pre-colonial Nigerian socio-economic systems, women were positioned for less enterprise and low-income business activities in the economy. This marginalization soon became a way of life in Nigeria and in Africa in general (Adu, 2013; Okoronkwo-Chukwu, 2013).

Upon the final exit of the British colonizers from Nigeria in 1960, men took over the various offices created and relinquished the low paid jobs they occupied during the colonial regime to women who had struggled to attain some educational level (Adu, 2013). Since then, lower cadre and less positioned jobs and businesses have become stereotyped as belonging to women. This, to date, has created segregation in the types of businesses and trades that woman can carry out.

This imbalance in the social structure has a negative effect on national development because the colonizers reinforced the patriarchal ideology that is now entrenched in the Nigerian society. This ideology creates the divide that keeps women at home, in the role of home makers, full-time housewives and or in petty trades and low profit-making businesses which inhibit them from utilizing their full potentials and participating fully in entrepreneurial activities. This results in a huge loss to the nation's economic development (Jaiyeola and Aladegbola, 2020). Besides, the patriarchal society forbids women to aspire and rise to greater roles as men do in business and other areas of their lives. This practice is common in Africa, and unfortunately women also reproduce this inequality because it is entrenched in the society that women have limitations in what they can do. This is pronounced in every sector of the business world, either small or medium, and all the essential requirements that facilitate the establishment and or development of entrepreneurial skills of women.

The stereotyping and stratification of businesses have become deeply rooted in Nigeria because of the cultures and ideologies of the society (Adu, 2013). Women face a lot of difficult situations and meet a lot of obstacles in turning their skills into monetary gains (Tende, 2016) even when the money is for the development and sustenance of the family and home.

However, many literatures identify various barriers to women entrepreneurs globally, which include imbalance between business and domestic duties, lack of family support, little or no access to bank loans and more (Tende, 2016; Raghuvanshi et al., 2017; Kitching and Woldie, 2004). But there are some cultural and geographical barriers that are peculiar to some regions. For example, in most African countries, women do not have inheritance rights to landed property which serve as collaterals for bank loans, nor do they have startup financial support from family because the families give such privileges to male children (Adu, 2013).

In addition, glass ceilings, low paying jobs, and poor profit-making businesses which contribute to most women being in poverty are characteristic of a typical African woman struggling to make a living.

In view of ameliorating this menace, many schools of thoughts including the United Nations organizations, scholars and feminists' groups believe that empowering women has become an indispensable tool for eradicating the gap existing between men and women and building women's capacity for self-development and poverty reduction. This will also contribute to improving the economic situation of women and indirectly increase the productivity of families, communities, and the nation. It will also improve the prospects of many generations to come. The importance of gender equality and women's empowerment is underscored by its inclusion in the United Nations' Sustainable Development Goals (SDGs) (United Nations, 2006).

Howbeit, women's empowerment is supposed to enhance women's participation in economic development by providing them with micro finance loans for startup and for improving their enterprises; assisting them in skill training and acquisition to gain skills useful for the establishment of trades such as fashion designing, food vending, cloth making, pottery and others which relate to their community and match their interest; or equipping them with modern equipment to facilitate quick and better production of their wares in their existing businesses, such as the women in Ogotun Ekiti who were involved in mat weaving and pottery. Unfortunately, empowerment programs and processes have been impacted by men, who dominate and hold power in every level of government. Therefore, much of the benefits are either misappropriated to fellow men or used for political interests. This often leaves women unfulfilled and in constant struggle to break through the barriers.

### 2.2 Theoretical framework

This study engages the Africa feminism framework for analysis. However, African feminism is split into two categories, namely the intellectual and popular African feminism (Atanga, 2013; Sachikonye, 2010). The intellectual feminists are the pragmatic African feminists who try to redefine women's role to make them more relevant in the social, political, and economic systems (Sachikonye, 2010). They advocate for an active voice for women against the taboos of gender hierarchy and the ancillary status of women. The intellectual feminist category rejects the culturally based practices affecting women such as female genital mutilations, girls and women's early marriages and lack of education, traditional gender roles and other subordinate roles. They embrace the abolition of these cultural practices by approaching African women's issues in a more pragmatic way, asking for women's rights and changes in policies to give women the right to ownerships and choice to participate in all areas of life (Sachikonye, 2010). The intellectual African feminism includes STIWANISM, which is the framework used for this study. Its main aim is to have a transformed African society that is not disposed to traditions that cripple a group of people and subject them to subordination either by religion or culture. It also seeks to transform the society through empowering women and girls to have equal opportunities as men and boys. This is very significant to this study with its focus on creating an enabling environment through a change of systems that will no longer keep women subjugated with traditions. White colonialism reinforced patriarchal dominance, and the days after colonial regimes have not been free from the same practice. The economic space has become a treacherous one for women entrenched by neo-colonial activities that oppress women, relegate them to the background in the society, and often place men at the top of the social ladder. The transformation of the African society is the responsibility of both men and women, and it is a collective interest (Akali et al., 2013). Ogundipe-Leslie argues that, historically, women have been kept from social development, and now is the appropriate time for African women to struggle to liberate themselves. But she was also concerned about the perception of African women about the existing structures, rooted in the culture of inequalities and power dominance of privileged men which women have taken as the preferred way of life. African women have lived with this pattern for a long time and have become dependent on men. This is evident in the ways women conduct their economic activities and record very low profit compared to men. Women should disrupt the norm and aspire for a movement, structure, and practice which will include them in the social transformation of the society (Ogundipe-Leslie, 1994), entrepreneurial activities, and economic growth.

### 2.3 Methodology

To understand the barriers confronting the rural women contextually from the participants' perspectives, it is necessary to hear their stories and to understand their experiences through their activities in their natural setting (Creswell, 2014). There were twenty in-depth interviews at Ogotun Ekiti, and an average of thirty minutes was spent with each participant. There were also two participants-observations, each lasting for twenty-five minutes, and four focus group discussions. There was a review and analysis of copies of historical records of a cooperative society's loans and documents, and few mats woven by the women served as artifacts. The literature and reports of the Ogotun Mat Weaving Empowerment Center, named Better Life for Rural Women, that were available online did not represent the real situation when the site was visited. The location had changed, and the operations of the government sponsored empowerment programs had been suspended. The choice of this location is basically because of the grassroots empowerment program, in form of a center called Ogotun Ekiti Mat Weaving Center, founded under the Better Life for Rural Women program. This center as well as its products was in the news some years ago. This location was also chosen because there are more women of low social economic status in the rural areas working to make a good business out of their skills and traditional trade than in the urban grassroots empowerment programs. It is also a convenient sample since one of the researchers is close to the gate keeper who could link us directly with the rural women without much protocol. The participants were mainly women between ages 35 and 80, and most of them were self-employed, particularly engaged in mat weaving business. We did some preliminary work to identify the key players prior to arriving the site of the research. The Institutional review board (IRB) at Washington State University demanded to have all the copies of documents to use with participants translated to Yoruba for review this was done. An exemption category was approved and obtained since the interviews and focus groups were for women of 18 years and above. Likewise, information about the projects, the consent forms and interview protocols were written and translated into local language (Yoruba) for participants understanding and approval before the project commenced. All approvals from Ogotun were verbal and documented appropriately.

### 2.4 Data analysis procedure

This is a process where meaningful patterns and themes emerged through thorough examination, comparison, contrasting and interpreting of the data collected (Miles et al., 2014). Meaningful patterns and themes were established consciously using the research questions. The first step in the data reduction process was the transcription of the interview data. Since more than half of my participants spoke in Yoruba, the next step was to translate the transcripts into English Language. This was followed by weeks of manual coding to get categories and subcategories. There were many sessions of brainstorming and writing, coding, and recoding, listening to tapes, rereading of the transcripts, and marking of the data with different colors. We worked together as a team and engaged the inputs of qualitative research experts and colleagues in the process. At the initial stage there were fourteen themes, which we considered large, but by using a deductive analysis approach four more focused categories and subcategories emerged. Finally, these four themes were further collapsed into three themes.

We kept an analytic memo that contained a first-hand report about the research process, especially throughout data collection and analysis. We have included substantial parts of the memo, and it has helped to piece together many data that cut across many concepts and topics coming up in the findings. Pseudonyms are used in writing these findings.

## 3 Findings and discussions

### 3.1 Theme one: patriarchal traditions obstructing women entrepreneurship

The findings suggest that culture and tradition play a vital role in the entrepreneurial activities of women at the grassroots level at Ogotun, as shown in the responses of the women about the attitude of men towards women. Ogotun men are a representation of Nigerian and African men, who enjoy the power and privileges of patriarchy. The women in the study share the same views about men.

#### 3.1.1 Men are seen as ‘lords’

This is a response a woman gave in her experience with the father of the house. The father is the landlord {(sic) way to talk about a man who gives orders on everything, these are the characteristics of most men} and the all-in-all in the house. Anything that he says is the final, so they don't listen to advice of women as such. They will want them to know that they are the leaders of that family. So, they don't even call women into their decisions (Debby, individual interview, May 27, 2019 at Ogotun Ekiti).

Men force women to follow them always to their (men) own farm and work for them and abandoning their own trade.

Women are subjected to such a condition that whenever they need money, they will have to beg, beg and beg the man until he will give it to her. (Debby, individual interview, May 27, 2019 at Ogotun Ekiti).

The landlord, most times, relates with ‘tenants’ under some stringent conditions. The women hardly get any financial support from their husbands after their bride price had been paid, thus denying them opportunities of engaging in any form of business. Men do not provide enough food and sometimes, none; they would rather make the women work on their farms, taking it as the men's traditional right as the owners of women. The men, as landlords, are capitalists who are always interested in profits and benefit from their properties or goods. Women, culturally, are regarded by men as goods, and they use them as such in terms of the production of food, children, sex, and care for the man.

#### 3.1.2 Men deprive women of their loans thereby stopping them from engaging in business

Women were organized into cooperative societies for easy access to loan assistance and repayment. This initiative was to help the women execute their mat weaving business and ensure that the women used the money for the appropriate purposes because their husbands sometimes forcefully take the money from them.

some men will collect that money(loan) from their wives, and it is the woman that will find a means to work in order to repay the money, they(men) will not pay it (Lydia, Focus Group Discussant, May 27, 2019 at Ogotun Ekiti).

#### 3.1.3 Men do not want their wives to engage in any entrepreneurial activities

There is no man that wants his wife to be self-independent. Men … do not want their wives to have freedom; neither do they want a woman to be the sole controller of a business, they do not want it… (Lydia, personal communication, May 23, 2019 at Ogotun Ekiti).

Men don't want their wives to work and earn money for themselves. She is, most times, not allowed to learn any skills or use her skills to make a living for herself to make her life better. (Debby, individual interview, May 27, 2019 at Ogotun Ekiti).

That is the type of woman the man and society/tradition regard as a submissive and responsible (good) wife (Debby, individual interview, May 27, 2019 at Ogotun Ekiti).

Debby believes that men do not want their wives to engage in any entrepreneurial activities. In another interview Sade reiterated this condition that:

some men because of their ego … certain percentage of men in the society because they prefer to put their wives under them (oppress), they deny women from having means of income or liberty to spend at their own. So that the woman will be coming anytime she needs something, she should be coming to plead but that is not the right thing (Sade, individual interview, May 30, 2019 at Ilawe Ekiti).

#### 3.1.4 Men use cultural practices as weapons to weaken women progressive activities

Men use cultural practices as social, psychological, political, and emotional weapons to make women see themselves as weak objects of subordination and fear, and as such, turn them to victims (Idowu, 2013).

A woman told me that whenever she wanted to go to the market for her trade was the day her husband will forcefully demand her to follow him to the farm. The woman would always suspend her plans and follow the man to the farm, she said, because if she did not go to the farm as demanded, she won't be allowed into the house again (Lydia, personal communication, May 23,2019 at Ogotun Ekiti).

### 3.2 Theme two: lack of government support in women entrepreneurial activities at the grassroots

The experience of women at Ogotun revealed that the role of government has been gradually diminishing in the support of grassroots entrepreneurial activities of women since the rule of the civilian government in 1999. According to Lydia, there is no significant impact of the civilian government on women's empowerment programs compared to the military government. She said:

It was the last military regime of Governor Bode George in the old

Ondo state government, through another Non-Governmental Organization (Country Women Association of Nigeria (COWAN) that gave loans to women then, though they were unable to do nothing more than that*.* But “Since the advent of civilian government, we have seen nothing” (Rewriitten for clearer understanding from translation). (Lydia, Focus Group Discussant, May 27, 2019 at Ogotun Ekiti)*.*

Lydia said the situation has changed since the civilian government came into power in 1999. The civilian government choose to give money to selected women who belong to their political party.

it is only those that are involved in politics that can obtain something from government … So it is only those that are closer to them that will receive something (money) from them (Debby, individual interview, May 27, 2019 at Ogotun Ekiti).

A man on the street of Ogotun talked of the role of government in women's empowerment towards entrepreneurship in the community. He said sometimes when money is involved, the process of collecting such money is cumbersome for women. They would have to travel, provide so many documents, and appear before different sets of people several times before the money is given out. Most times they would have spent about two-thirds of the expected money on processing and travels, so that the little left for the business purpose would not give them the anticipated profit. This is far different from the easy loan systems facilitated under the military administration through cooperative societies.

This was confirmed in an interview session with the deputy director of the community development department at the local government office. He said:

they (representatives of the State government) will invite the community department director, to present the list of twenty women and it should cut across the three towns in the local government, the government wants to give them financial assistance, grants or loan. When that is done, such nominees will go directly to Ado Ekiti (State capital) to get their loans, not coming here (local government office), they will process all their forms, they will take them to Ado Ekiti at the Bank which they will transact the business; they will open the account and do it there (Jide, Focus Group Discussant, May 30, 2019 at Ilawe Ekiti).

The loan processing procedure is considered cumbersome for the rural women as they usually have to spend much of their time as well as much money from their little resources. During the focus group discussions, the women at Ogotun said the government abandoned the promotion of their crafts and destroyed the legacy that the military administration gave them in empowering them through their mat weaving. It is sad to note that the military government could be more responsive to women's need than ‘the government of the people’.

In their stories, the Ogotun women said the military government built a center for empowerment in their community where they go to weave mats. They were paid for the mats, and sometimes, the mats were bought at the center and used to make other products. This center was called “Better Life for Rural Women” and was equipped and staffed by the local government. The center used different machines to produce bags, table mats, covers, folders and other household materials from the woven mats. Better Life for Rural Women was an initiative by Maryam Babangida (wife of a former military president) to provide empowerment for rural women in Nigeria in 1987. She proposed this at the grassroots level hence its name, rural women. This was done through agriculture and cottage industries like the mat weaving in Ogotun. The center in Ogotun thrived under the military rule and a little after then, according to the women. The women confirmed that the government and the women were making profit from the center. Unfortunately, the women lamented the sale of the center after it had been abandoned for several years under the civilian rule.

the Better Life for Rural Women (training center) has been sold by the government… Ah! We people working there didn't understand. Government from council owns the place and what pleases an owner, he will do with what he has. They were the ones that sold their property. Even, we people working there were not informed when the council sold it (Rose, Focus Group Discussant, May 27, 2019 at Ogotun Ekiti).

### 3.3 Theme three: Women's lack of financial support for entrepreneurship

The women in this study resorted to various self-help strategies to overcome their financial barriers when they acknowledged their deprivation, oppressions, and inability to access financial resources. Since colonialism and its accompanying patriarchy forced them out of their former profession of cash crop farming (and men took over) (Ako-Nai, 2013), and the assistance of government and development agencies were not forthcoming to them, these women, therefore, turned to self-help strategies to support themselves and meet their economic needs. They used two main strategies to approach their self-help. These are collective and individual strategies.

### 3.4 Collective efforts

A collective approach was employed by the women to raise funds and loans among themselves. They pooled their meagre resources together to help themselves and launched a private society called Ifedapo meaning “love has joined us together.”

Each member makes a daily contribution of twenty naira ($.0055), which represents a daily savings (Esusu) for these women, but when these amounts are put together for every member, it is substantial. This is a strategy of a locally organized micro credit system. Some private entrepreneurs and banks have an improved system of this approach called micro finance banks. This is common in some developing countries like Nigeria, Bangladesh, and India. Micro finance banks are established to help the poor finance their businesses; however, in recent times, they charge high interest rates to meet their overhead cost and services though they claim that they are not set up for profit making. This high interest rate and excessive bank charges, in some cases, are barriers to the rural women (Kanu and Idume, 2015) to access such funds. However, the women in Ifedapo society were not organized after this pattern; rather, the goal of the society is to support very needy group members by providing loans without any interest or service charge. Tam said whoever needed money is given a loan without interest from the group's money.

I used it for two years to work on my mat plantation and after those two years my mat farm that I used it for increased. I earned more money and I never went hungry again (Tam, personal interview, May 27,2019 at Ogotun Ekiti).

This was confirmed by other women in the focus group discussions who spoke on how they made use of the money they got from their society.

We used it to clear the bush, we would hire the service of laborers to clear the farmland and prepare it. The land then becomes fertile for the mat trees to flourish. We are always excited after a time to see that the leaves were appearing fresh. Then we pray that it will be well with those that borrowed us this money, they have done something nice. We see that the mat trees have grown so well and increased in quality.

### 3.5 Individual efforts

Besides the collective efforts, these women work and employ different strategies, individually, to support their entrepreneurial activities since the support of the collective group is not usually enough to meet their needs. Most of the women engage in farming, while others engage in trading to get enough money. For example, Olu, a woman between 55 and 60 years old, said that her husband would not give her money for business. *“*I am the one that sponsors myself.” She remarked that it is a difficult thing for men to support their wives in business because of their selfish interest.

Ahh, No one gives me money; it is God that gives me money for my business, (laughing). It is God that gives me money for business. I engage in no other sales than these mats. I do my business alone*.* No support from my husband. (Rose, 80 years old but very agile). She continued:

whenever I go to the farm, whatsoever produce I find there, or if it was mats, I will sell it and use the money to hire workmen (nomadic, not the local men, from another small tribe search for such farm work to sustain their families and they often settle in small communities to be hired as farm labors) to do my farm work. I am the one doing it all. I usually assign someone to the farm and come back home to continue my mats weaving. I have money to do farm work and, at the same time, take care of my needs. (rewritten for the understanding of the English reader)

Sometimes these women combined their efforts and ideas to work and help themselves in their mat weaving to have quick turnovers and meet buyers’ specifications. Such was the experience Olu shared with me:

Mat weaving is quite innovative, some of us only know how to weave white mats while some know how to weave red mats. They are of several types. There are some mats that are of different patterns, while some are with white patterns while some are mixture of the patterns. But then there are some of us that know how to weave all the types of mats, so we go to meet such people that know better on the different types of the mats weaving and discuss the specifications that is given by a potential buyer and …we do it together and share the money (Olu, individual interview, May 27, 2019 at Ogotun Ekiti).

These are some of the strategies these women used to work around the challenges, and they have good outcomes for their efforts**.**

Recounting the benefits from their self-support systems, one of the women said:

when I started weaving mats of different designs, I sold them and whenever my children asked for money from me, I gave them. (Rose, individual interview, May 27, 2019).

However, as much as the efforts of these women to overcome their hindrances and challenges are yielding positive results, they still feel the burden and hardship badly, as noted by one of the respondents:

it is a heavy load on those women because they don't sleep at night. They will do the mat work throughout the night and when it is morning, they will follow their husbands to farm. Those women are usually overworked, and this could make them become aged quickly. Except sometimes when their husbands do not have work on the farm, they would stay home to do their own mats in the day but it is not often so (Debby, personal interview, May 27, 2019 at Ogotun Ekiti).

## 4 Implications and recommendations

### 4.1 The implication for government

The current government bureaucracies in Nigeria are detrimental to the entrepreneurial activities of women at the grassroots. Constitutionally, community development is the duty of the local government in Nigeria (Khemani, 2001); the local government brings governance and development to rural regions through the funding of projects and programs, and rural women are expected to benefit from such an arrangement. The Better Life for Rural Women Center at Ogotun is an example of such initiatives when it was in its full operational state. Unfortunately, the state government controls all its funds, and this makes it difficult for the local government to fund local initiatives to support its constituents. For example, whenever an organization gives grants to fund women's projects and ideas, it is often administered through the state government without the input of the local government. Another issue is that donors and agencies work in collaboration with the politicians in government who would only give financial aids to their supporters in the urban areas thereby leaving the rural women in impoverished states. Therefore, to resolve these bureaucratic policies and processes, the local government should be given autonomy to carry out its statutory functions, as stated in the Nigerian constitution, towards community development and made accountable to the people. That is democracy, power must be in the hands of the people.

In addition, government officials at all levels need to be educated about the predicament of rural women engaged in entrepreneurial activities, so they can take adequate measures to ease their sufferings. Media has a key role to play in publicizing women's situation. Government officials should continually reassess the situation of women to formulate policies and install a management system that will support their financial needs and enable them to access interest-free loans without bottlenecks. The government will help women entrepreneurship by incorporating entrepreneurial knowledge through Knowledge-Intensive Business Services (KIBS) and providing funds generously for innovative research so women entrepreneurs can receive support and education for their enterprise; this will also help new entrepreneurs (Adeyeye et al., 2019).

### 4.2 The implication for educators to work in the community

Education is empowerment; it produces knowledge. Therefore, various levels of educators, researchers and program funders should collaborate with the community development departments for public enlightenment programs to provide good informative sessions about the issues of the wellbeing of women and change the perspectives of the people. This is necessary because both men and women need to be educated about the effect of cultural practices that oppress women. Their marginalization and deprivation of rights is a waste of human resources. It gives rise to premature aging in both men and women because of the associated stress. Moreover, with the marginalization of women, life becomes one-sided because nature created men and women to complement each other.

This critical education will create awareness and understanding in the community that may lead to the liberation of women from dominant cultural beliefs and practices and give them the quality lives they choose to live. They would also be able to make profit in their businesses. This is a sure road to gender equality - a target of United Nations by 2030 (UN, 2015).

### 4.3 Implications for funders of women programs

The local and international organizations which advocate for gender equality and women development should make women's empowerment the focus in their various intervention programs and projects. This will enable women to overcome some of the obstacles highlighted in this research. The support and intervention may be in form of training, provision of resources, loans without collateral, grants, market information, and adequate and updated equipment. The key players among these funders include the United Nations (UN), United States Agency for International Development (USAID) and other international development agencies and non-government organizations (NGO). However, the leaders of these organizations need to know and understand the peculiar experiences of rural and urban women at the grassroots level in the locations they serve (in this case the Ogotun women serve as an example of women in rural southwestern Nigeria), as the same cap may not fit all heads. The big donors also need proper guidelines on how to operate with intermediary and government agencies who represent their interest. Consequently, the donors would be able to address the needs of the rural women and overcome their obstacles by channeling funds towards grassroots support and women empowerment, as it is obvious that rural women entrepreneurial activities are deteriorating.

## 5 Conclusion

This research paper has demonstrated through the case study analysis of the rural women in Ogotun involved in mat weaving business that African women are endowed with skills and the spirit of entrepreneurship. This was also highlighted from the literature dating back to the pre-colonial era. However, in this 21st century, women in Africa are confronted with various obstacles that thwart their laudable efforts as shown in this study. This was attested to by the World Bank's assessment of women in entrepreneurship in Africa which placed women at the lower deck of economic breakthrough in comparison to men. Although both men and women operate in the same business climate, women are usually poorer and are harder hit by adverse economic situations because their efforts are usually met with huge obstacles and barriers. Data were collected and analyzed through qualitative case study research design, and three themes emerged as the final findings.

The findings reveal the dynamics of many factors that are embedded in patriarchy that form the foundation of oppression of women by men. They also depict the re-inscription and reproduction of these oppressions by the government institutions expected to salvage the situation of women. However, the self-help projects of the women in this study were a step towards empowerment by women, without the input of men nor the government, to improve their businesses. Women have demonstrated through their successful self-empowerment strategies that it is possible to attain the heights they want if the barriers of patriarchy, unresponsive government and poor accessibility to external funding are eliminated. Women have the business acumen, spirit, and resources to raise the bar of the economy if these identified obstacles can be removed.

## Declarations

### Author contribution statement

Mercy Modupe Adeyeye: Performed the experiments.

Emmanuel Femi Jaiyeola: Conceived and designed the experiments; Analyzed and interpreted the data; Wrote the paper.

### Funding statement

This research did not receive any specific grant from funding agencies in the public, commercial, or not-for-profit sectors.

### Data availability statement

Data included in article/supplementary material/referenced in article.

### Declaration of interests statement

The authors declare no conflict of interest.

### Additional information

No additional information is available for this paper.
